# Genetic complexity alters drug susceptibility of asexual and gametocyte stages of *Plasmodium falciparum* to antimalarial candidates

**DOI:** 10.1128/aac.01291-23

**Published:** 2024-01-23

**Authors:** Nicola Greyling, Mariëtte van der Watt, Hazel Gwarinda, Ashleigh van Heerden, Bryan Greenhouse, Didier Leroy, Jandeli Niemand, Lyn-Marié Birkholtz

**Affiliations:** 1 Department of Biochemistry, Genetics and Microbiology, University of Pretoria, Pretoria, South Africa; 2 Institute for Sustainable Malaria Control, University of Pretoria, Pretoria, South Africa; 3 Department of Medicine, University of California-San Francisco, San Francisco, California, USA; 4 Medicines for Malaria Venture, Geneva, Switzerland; The Children's Hospital of Philadelphia, Philadelphia, Pennsylvania, USA

**Keywords:** *Plasmodium falciparum*, clinical isolates, gametocytes, differential compound sensitivity, genetic diversity, malaria

## Abstract

Malaria elimination requires interventions able to target both the asexual blood stage (ABS) parasites and transmissible gametocyte stages of *Plasmodium falciparum*. Lead antimalarial candidates are evaluated against clinical isolates to address key concerns regarding efficacy and to confirm that the current, circulating parasites from endemic regions lack resistance against these candidates. While this has largely been performed on ABS parasites, limited data are available on the transmission-blocking efficacy of compounds with multistage activity. Here, we evaluated the efficacy of lead antimalarial candidates against both ABS parasites and late-stage gametocytes side-by-side, against clinical *P. falciparum* isolates from southern Africa. We additionally correlated drug efficacy to the genetic diversity of the clinical isolates as determined with a panel of well-characterized, genome-spanning microsatellite markers. Our data indicate varying sensitivities of the isolates to key antimalarial candidates, both for ABS parasites and gametocyte stages. While ABS parasites were efficiently killed, irrespective of genetic complexity, antimalarial candidates lost some gametocytocidal efficacy when the gametocytes originated from genetically complex, multiple-clone infections. This suggests a fitness benefit to multiclone isolates to sustain transmission and reduce drug susceptibility. In conclusion, this is the first study to investigate the efficacy of antimalarial candidates on both ABS parasites and gametocytes from *P. falciparum* clinical isolates where the influence of parasite genetic complexity is highlighted, ultimately aiding the malaria elimination agenda.

## INTRODUCTION

Malaria remains one of the most prominent infectious diseases, with 95% of cases occurring in the African region as defined by the World Health Organization. *Plasmodium* spp. are the causative agents of malaria, vectored by *Anopheles* mosquitoes, and accounted for an estimated 247 million cases of malaria across the globe in 2021, with 619,000 deaths ascribed to the disease (1). The complex life cycle of *Plasmodium* parasites (2) supports replication during asexual blood stage (ABS) development, leading to parasite population expansion, disease-associated pathology, and sexual reproduction after formation of male and female gametocytes in the human hosts (3). These developing gametocytes of *Plasmodium falciparum* are morphologically and functionally distinct from ABS parasites (4–6), with a uniquely extended maturation process of 8–12 days. Only 10^3^ mature, stage V gametocytes are required to sustain transmission to the *Anopheles* mosquito, which makes this population bottleneck an attractive target for transmission-blocking interventions (7).

With the circumvention of antimalarial drug resistance development and spread remaining a key focus for drug discovery campaigns, any new compounds are required to kill multiple stages of the parasite while targeting novel biological processes (8–10). Several lead clinical candidates in development by the Medicines for Malaria Venture (www.mmv.org) and associated partners have multistage activity against ABS parasites and gametocytes (8, 9), including Cipargamin (KAE609, with Novartis), Ganaplacide (KAF156, with Novartis), and Cabamiquine (M5717/DDD498, with Merck KGaA). As part of the discovery process, hit compounds with *in vitro* activity against the parasites are typically also evaluated for efficacy against multiple laboratory-adapted *P. falciparum* strains (11) with various drug resistance genotypes and phenotypes (12), but importantly also against ABS parasites of clinical isolates from field samples (12–16). This provides an indication of variant efficacies against circulating parasite populations in malaria-endemic regions to ensure resistance is unlikely to exist or rapidly emerge. Additional genotypic profiles of these clinical isolates are used to provide supportive information on genetic polymorphisms in known drug resistance alleles and drug targets (16, 17).

We previously reported extension of such drug efficacy evaluations on clinical isolates to additional life cycle stages of the parasite. We specifically evaluated the gametocytocidal activity on southern Africa *ex vivo* isolates that were able to produce gametocytes *in vitro* (18). This provided key information required to profile a candidate for multistage activity, an important goal for transmission-blocking activity (8). Although the majority of the compounds retained activity on the clinical isolates, differential drug responses were observed for several of the kinase inhibitors evaluated (18). Here, we extend this to evaluate the *ex vivo* gametocytocidal and ABS action of front-runner and lead antimalarial candidates on 11 clinical isolates of *P. falciparum* from southern Africa. We link the stage-specific or multistage susceptibility of the clinical isolates to these candidates to drug resistance markers but importantly also to extended genotyping. This allowed us to correlate the genetic complexity of the clinical isolates to their drug susceptibility. By providing early indications of antimalarial compound *ex vivo* efficacy, these isolates have the potential to aid in the screening of compounds with transmission-blocking activity, thereby aiding in the malaria elimination agenda.

## MATERIALS AND METHODS

### Participant consent

Isolates were sampled between February and April 2014 from patients ≥ 21 years of age who presented with a positive malaria diagnosis {blood smear or *P. falciparum* sero-positive [First Response Malaria Antigen *P. falciparum* (HRP2) Detection Rapid Card Tests] and symptomatic} at either the Steve Biko Academic Hospital, Tshwane District Hospital, or Kalafong Hospital in Gauteng Province, South Africa. The presence of mixed *Plasmodium* species infection was ruled out microscopically. Patient identity was blinded, but age, gender, place of origin (and travel history), and presenting percent parasitemia at diagnosis and available prophylaxis history was captured. Samples were taken before any antimalarial treatment was initiated. Patients were subsequently treated for malaria with antimalarials as per the South African National Department of Health guidelines in the Division of Infectious Diseases, Department of Internal Medicine.

### Evaluation of the asexual stages of *P. falciparum* clinical isolates

*Ex vivo* cultures (i.e., cultures where the parasites were not allowed to adapt to *in vitro* cultivation conditions and therefore reflect minimal alterations) were initiated from 11 clinical isolates within 2–8 h after intravenous blood collection and adaptation was limited to a maximum of five passages (full 48 h asexual replication cycles) before gametocyte induction was performed, to limit culture adaptation and potential genotype changes (18). Cryopreserved stocks were prepared at the initial culture initiation to preserve possible polyclonality of the *ex vivo* cultures from which all subsequent gametocyte inductions were performed. *P. falciparum* laboratory-adapted strain NF54 (PfNF54) was obtained from MR4 (www.beiresources.org). Isolates and PfNF54 were cultured *in vitro* in human erythrocytes (5% hematocrit) in complete medium [RPMI-1640 with 23.81 mM sodium bicarbonate, pH of 7.4; 0.5% (wt/vol) Albumax II, 80 mg/mL gentamycin, 25 mM HEPES, 20 mM glucose, and 0.2 mM hypoxanthine] at 3-5% parasitemia, 37°C, shaking at 60 rpm under hypoxic conditions (90% N_2_, 5% O_2_ and 5% CO_2_). Medium was replaced daily, and parasite morphology and proliferation were monitored by Giemsa-stained slides visualized with light microscopy using a ×100 oil immersion lens at a ×1,000 magnification.

### Drug resistance gene indications

All the isolates were genotyped for drug resistance against artemisinin (ART), chloroquine (CQ), dihydroartemisinin (DHA), mefloquine (MQ), pyrimethamine, and amodiaquine (AQ) as previously described (18). PCR and restriction fragment length polymorphism for asexual drug resistance markers *dhfr* (codons 50, 51, 59, 108, and 164), *dhps* (codons 436, 437, 540, and 581), *pfcrt* (codon 76), and *pfmdr1* (codon 86) were performed (14).

### Production of viable *P. falciparum* gametocytes from clinical isolates

Gametocytogenesis was induced as described in reference (19) after one ABS cycle (48 h in culture). Briefly, ring-stage intraerythrocytic *P. falciparum* parasites (5% hematocrit, 5% parasitemia) were synchronized using 5% (wt/vol) D-sorbitol for 15 min at 37°C and were used to initiate gametocytogenesis, with the hematocrit and parasitemia adjusted to 6% and 0.5%, respectively. The parasites were transferred to a glucose-deprived medium to produce nutrient-starved and stressed parasites as described (20). After 72 h, the hematocrit was reduced to 4% and gametocytes were cultured for 5 days in glucose-deprived medium, and the remaining days in glucose-supplemented medium under hypoxic conditions, stationary at 37°C and monitored daily with Giemsa-stained microscopy (20). Elimination of asexual parasites was achieved with 50 mM N-acetyl-glucosamine treatment for 6–9 days post-induction (20, 21). On day 11, the gametocytemia and stage distribution of the cultures were evaluated as above.

### Gametocyte viability indications

Late-stage gametocyte (>95% stage IV and V gametocytes, 67% stage IV, and 29% stage V) viability was first evaluated using confocal fluorescent microscopy with hydroethidine (HE) as viability stain. Parasites were stained with 5 µM HE for 2 h at 37°C, fixed overnight with 0.025% (vol/vol) glutaraldehyde/phosphate-buffered saline at 4˚C. An HeNe laser with a 543 nm wavelength was used to excite HE and emission captured on a Zeiss 510 META confocal laser scanning microscope (Zeiss, Germany) (19). A minimum of 10 images per *P. falciparum* isolate was taken.

The viability of late-stage (stage IV or V) gametocytes was determined with the parasite lactate dehydrogenase (pLDH) assay as described (20, 22), using 200 µL 2% gametocytemia, 1% hematocrit cultures, incubated for 48 h at 37°C. Malstat reagent [100 µL of 0.21% vol/vol Triton X-100; 222 mM L-(+)-lactic acid, 54.5 mM Tris, 0.166 mM 3-acetylpyridine-adenine-dinucleotide, pH 9] was added to 20 µL of parasite suspension, followed by addition of 1.96 mM nitro-blue tetrazolium and 0.0239mM phenazine ethosulfate. Plates were incubated in the dark for 40 min, after which absorbance was measured with a SpectraMax Paradigm Multimode Detection Platform (Molecular Devices) at 620 nm. The absorbance values of co-cultured erythrocytes were subtracted as background absorbance. Turnkey’s multiple comparison test was performed using GraphPad Prism version 9.5.1 (GraphPad Software Inc., La Jolla, CA, USA) to compare gametocyte viability.

### Susceptibility of ABS parasites and gametocytes from clinical isolates to antimalarial compounds

All the isolates were phenotyped for efficacy to ART, CQ, DHA, MQ, MB, and AQ against ABSs. In addition, a panel of six lead and interrogative antimalarial compounds active against both ABS and late-stage gametocytes (>95% stages IV andV) with different targets and mode of actions were selected to evaluate compound sensitivity of the clinical isolates compared to PfNF54. These compounds included four target-specific dual active compounds: MMV390048 (048) [targeting PfPI4K with some activity against PfPKG (13, 23)], SJ733 [targeting PfATP4 (23)], KAE609 [targeting PfATP4 (23, 24)], and M5717 [DDD498, targeting translation/elongation factor 2 (10, 25)]. Additionally, OZ439 was included as a compound without a specific protein target but with some involvement in hemoglobin metabolism and/or protein alkylation (10, 23, 26). Lastly, compound ML324 was included as it has preferential activity against gametocytes over ABS parasites and is proposed to target a Jumonji C domain-containing histone demethylase (27, 28).

Clinical isolate sensitivity to the selected compounds was determined for ABS using SYBR Green I fluorescence assays following 96 h incubation (29). Briefly, compounds were dissolved in a non-lethal dimethylsulfoxide (DMSO) concentration (<0.025%), serially diluted in culture medium, and added to ring stage intraerythrocytic *P. falciparum* parasites (1% parasitemia, 2% hematocrit), incubated at 37°C for 96 h in hypoxic conditions. Chloroquine (1 µM) was used as positive drug control for rapid, complete inhibition of parasite proliferation as a measure of the background fluorescence that is subtracted from all values. Parasite proliferation was normalized to the percentage of untreated control. Non-linear regression curves were generated using GraphPad Prism version 9.5.1, from which the half-maximal inhibitory concentrations (IC_50_) could be determined. For the gametocyte stages, a modified pLDH assay was used (20, 22) on day 11 of gametocyte induction when >95% stage IV and V gametocytes were visible, with a 72 h drug pressure, washout, and a further 48 h incubation period before determining pLDH activity. The IC_50_ values of the compounds were first confirmed against the PfNF54 lab-adapted strain, and subsequently, all clinical isolates were treated with the compounds at 2× IC_50_. Gametocytes from a select set of isolates were exposed to control antimalarial compounds for 48 h at 1 µM each. Assay platform robustness was evaluated by the *Z*′ factor for both ABS (30) and gametocyte assays (20). Data for known antimalarials were obtained on one independent biological repeat, while data for front-runner compounds are from three independent biological repeats, each performed in technical triplicates, as indicated. To quantify associations between *ex vivo* drug susceptibilities, we calculated bivariate correlations between median IC_50_ values using Spearman’s rank-order correlation coefficient to account for non-parametric distributions of IC_50_ values.

### Multilocus genotyping of clinical isolates using microsatellite analysis

A panel of 26 microsatellite (MS) markers spread across the *P. falciparum* genome (31) was used to genotype the clinical isolates as described before (32). The 26 MS loci included a panel of 16 loci that flank 10 MS markers (PolyA, Ta81, TA87, TA1, TA109, TA40, ARA2, pfPK2, PfG377, and TA60) that were found to be neutral and polymorphic. Genomic DNA was extracted from dried blood spots made on the day of isolate collection using the saponin-Chelex method (33). Two rounds of PCR were used to amplify the 26 MS loci for 9 of the 11 clinical isolates as described before (31, 32, 34). To automate the identification of true alleles and differentiate real peaks from artifacts, the electrograms obtained were analyzed using microSPAT software (https://github.com/EPPIcenter/MicroSPAT/wiki). Data were obtained in a form of allele sizes with varying base pairs per loci for each clinical isolate. To determine the number of genetically distinct parasite clones present in each isolate and to minimize overestimation, the multiplicity of infection (MOI) was calculated as the second-highest number of alleles detected at any of the 26 genotyped markers for each isolate (34). Analysis of variance pairwise *t*-test was used to compare the MOI between the nine isolates (32). To determine the level of outcrossing of clones within an individual infection, the Fws index (the within-host infection fixation index, indicating within-host genetic diversity of each isolate as inbreeding metric) was used. Fws ranges from 0 to 1 with a low Fws value indicating low inbreeding rates within the parasite population and thus high within-host diversity relative to the population. Thresholds of Fws of ≥0.95 identify isolates containing a single genotype (or “clonal” infections) and Fws of ≤0.70 indicate isolates with highly diverse infections (32, 35–37). Multiple correspondence analysis (MCA) was performed to identify isolates with similar genetic profiles using the Factoextra package in R for analysis of multiple categorical variables.

### Statistical analyses

Parasitemia (%), gametocytemia (%), and all activity data (IC_50_ or % inhibition) were determined from three independent biological repeats, each performed in technical triplicates to determine means with standard error, except where otherwise indicated. In these instances, unpaired, two-tailed Student’s *t*-test was applied to measure differences around the means. Data were analyzed using GraphPad Prism version 9.5.1. Pearson’s correlation coefficients were used to determine linear correlations between parasitemia and gametocytemia, as well as the relationship of these to genetic complexity with 1-Fws as indicator. To calculate relationships between within-host genetic variability (1-Fws) and drug efficacy (IC_50_ or % inhibition data), Spearman’s rank-order correlation was used to account for non-parametric data distributions with data analysis done using Python SciPy package (38).

## RESULTS

### Profiling the clinical isolates’ proliferation and gametocyte differentiation phenotypes of clinical isolates

**Fig 1:**
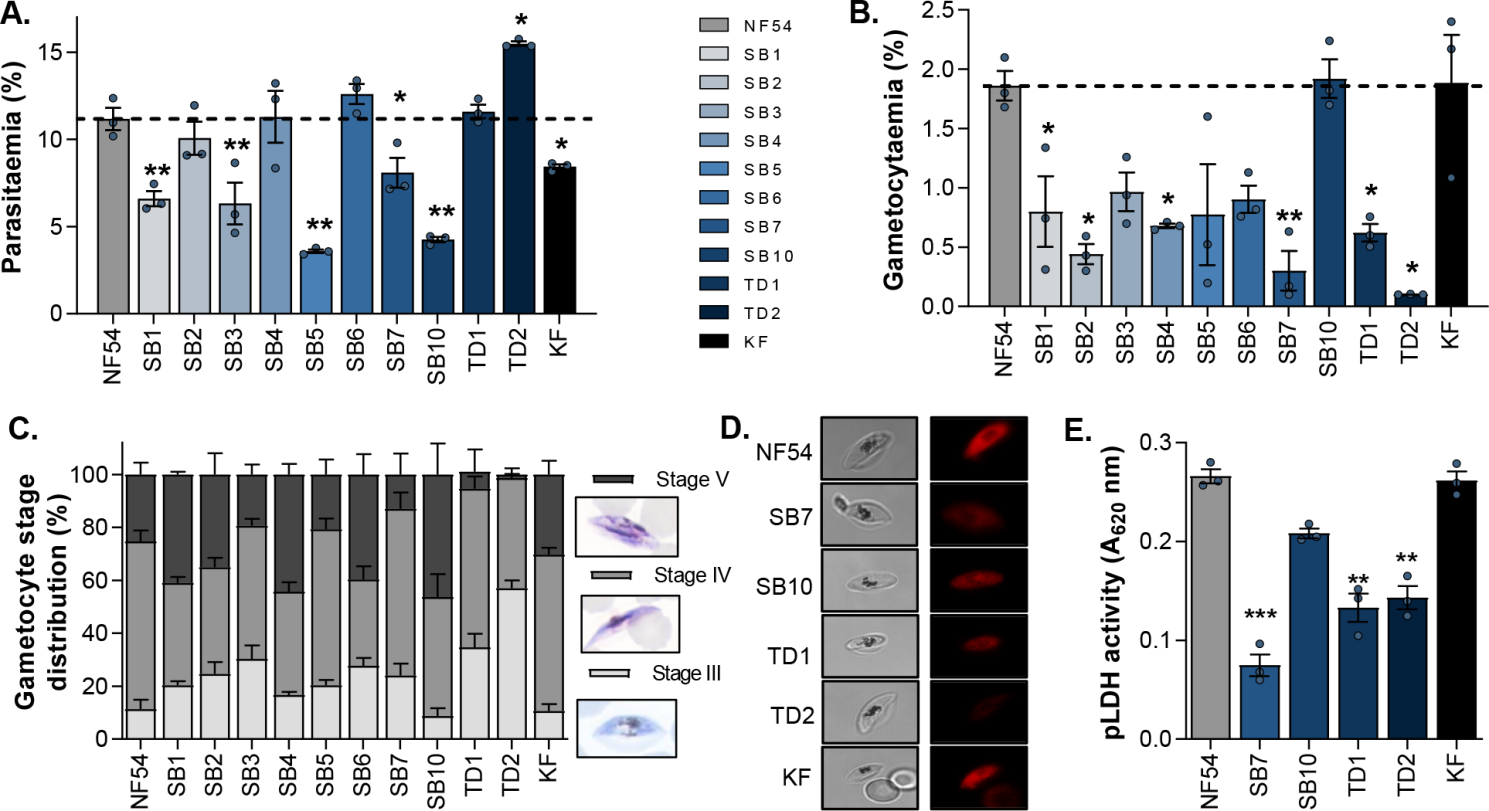
*P. falciparum* NF54 and clinical isolates’ ABS and late-stage gametocyte phenotypes. (A) ABS parasitemia after 96 h determined with a SYBR green I fluorescent assay.** (B)** Gametocytemia on day 11 after induction using Giemsa-stained slides. (C) Gametocyte stage distribution of clinical isolates and NF54 reference strain on day 11 of gametocytogenesis, representative Giemsa-stained stage III–V gametocytes. Late-stage gametocyte viability qualitatively evaluated using HE, with bright fluorescent red as indicator of live cells (**D**) or determined spectrophotometrically by measuring pLDH activity (**E**). In all instances, data are from three independent biological repeats (except for HE staining), with mean ± standard error indicated. Student’s *t*-test significance indicated as *P ≤ 0.05, **P ≤ 0.01, ***P ≤ 0.001, in comparison to NF54 reference values.

To compare the fitness of the clinical isolates to that of the lab-adapted *P. falciparum* reference strain, PfNF54, their ability to replicate and produce late-stage gametocytes for reproduction was determined. ABS parasite proliferation was monitored for 96 h from equal starting parasitemias of 1%. After the two proliferation cycles, several isolates (SB2, SB4, SB6, TD1, and TD2) displayed final parasitemias of ≥10%, like that obtained for the PfNF54 reference strain (Fig. 1A). TD2 was highly prolific, reaching parasitemias of almost 15% within two life cycles (replication factor of 4.9 ± 0.1), indicating that this isolate may have an *in vitro* replication fitness advantage compared to the other isolates and PfNF54. The remaining isolates had significantly lower parasitemias compared to PfNF54 after 96 h (*P* < 0.01, *n* = 3, *t*-test; Fig. 1A), with isolates such as SB5 and SB10 not even reaching 5% parasitemia, indicative of replication factors as low as 1.8 ± 0.03 and 1.5 ± 0.02, respectively. These isolates therefore reflect a more conservative population expansion during *ex vivo* proliferation compared to the clonal line PfNF54. Similar low multiplication rates have been previously reported for clinical malaria isolates during the first *ex vivo* cycle (39).

Most of the clinical isolates were poor gametocyte producers (Fig. 1B), with only isolates SB10 and KF producing similar mature-stage gametocytemias (~2%) to the PfNF54 reference strain. Some isolates (e.g., SB4, SB6, TD1, and TD2) that showed strong proliferation phenotypes were some of the weakest late-stage gametocyte producers. In fact, TD2 with the strongest ABS parasite proliferation phenotype was in essence unable to produce mature gametocytes (Fig. 1B). Overall, this translated into an anticorrelated relationship between parasitemia and gametocytemia (Pearson’s *r*^2^ = −0.37, Fig. S1), taking all the isolates into account. This points to preferential proliferation or differentiation phenotypes in the clinical isolates, where replication is prioritized above reproduction required for transmission.

To clarify the decreased gametocytogenesis in most clinical isolates, the isolates were monitored throughout the 14-day gametocytogenesis process. The low gametocytemias of isolates such as SB7, TD1, and TD2 were directly related to decreased production of stage IV and V gametocytes (Fig. 1C), with <10% stage V gametocytes detected for these isolates. The opposite was also true for isolates such as SB10 and KF, where more stage V gametocytes were already produced on day 11 of gametocytogenesis compared to strain PfNF54. For both these isolates, this directly translated to viable stage V gametocytes as indicated qualitatively with HE viability staining (Fig. 1D; Fig. S2) and quantified as decreased metabolic activity (via pLDH activity) (Fig. 1E) compared to the NF54 reference strain. This was not observed in the mature gametocyte populations for SB7, TD1, and TD2, with compromised gametocyte viability, that would influence the production of functional gametes during subsequent transmission steps. Therefore, most of the isolates were able to produce viable asexual parasites and gametocytes, although not unexpectedly, not to the same extent as the PfNF54 reference strain. However, there was a clear distinction between replicative vs reproductive preferences in the clinical isolates.

### Asexual *P. falciparum* clinical isolates display varying response to lead antimalarial candidates

**Fig 2:**
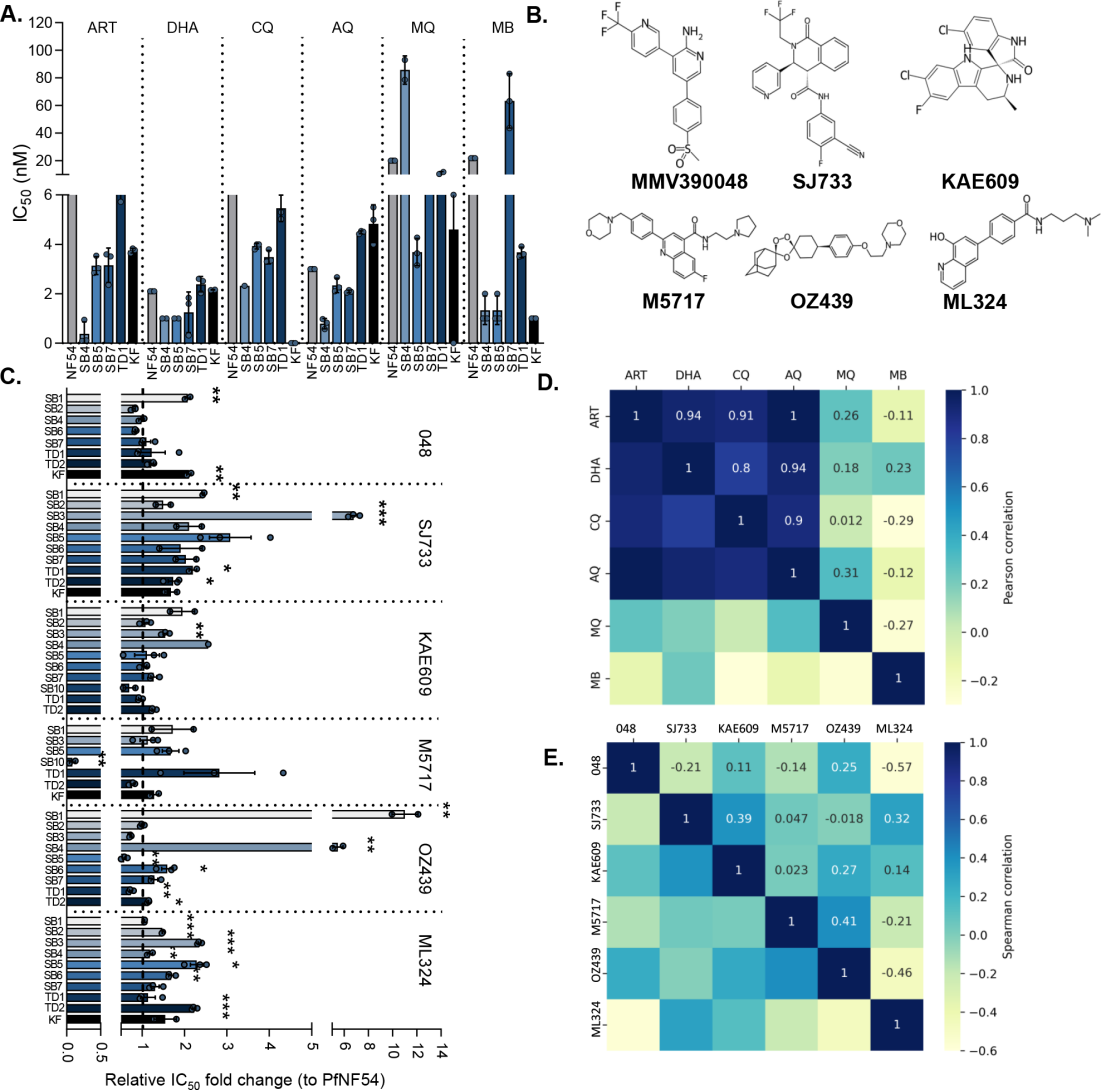
Variation in antimalarial IC_50_ sensitivities of *P. falciparum* ABS from clinical isolates. (A) Activity of standard antimalarial compounds [artemisinin (ART), dihydroartemisinin (DHA), chloroquine (CQ), amodiaquine (AQ), mefloquine (MQ), and methylene Blue (MB)] against the *P. falciparum* clinical isolates compared to the NF54 reference strain. Data are from a single biological experiment with technical triplicates, SD indicated. (**B)** Chemical structures of the six lead antimalarial candidates (MMV390048, SJ733, KAE 609, M5717, OZ439, and ML324) extracted from ChEMBL (https://www.ebi.ac.uk/chembl/). (**C)** Fold change in IC_50_ of the *P. falciparum* clinical isolates relative to PfNF54. Data are from three independent biological repeats, each performed in technical triplicates, mean ± standard error indicated. Student’s *t*-test significance indicated as *P ≤ 0.05, **P ≤ 0.01, ***P ≤ 0.001. Spearman’s rank correlations between the activities (IC_50_ values) among the known antimalarials (**D**) and lead candidates (**E**) are indicated in the correlation plots. The magnitude and direction of the association are indicated in each instance.

To provide an initial profile of the drug susceptibility of ABS parasites produced from the clinical isolates, these parasites were evaluated for their *ex vivo* sensitivity to known antimalarials (Fig. 2A). MB was included as an internal control and was also used to evaluate gametocytocidal activity. Assay quality parameters included *Z*-factors of >0.8, reflecting robust and reproducible evaluation of parasite proliferation (30), with the IC_50_values of all the known antimalarials tested against PfNF54 corresponding to previous reports for all compounds tested (Fig. S3; Table S1). All clinical isolates similarly exhibited nanomolar sensitivity to ART, DHA, and the 4-aminoquinolines (Fig. 2A). However, some loss of efficacy was seen for MQ and MB against ABS parasites from isolates SB4 and SB7 (Fig. 2A), although this loss was less than a threshold of >5-fold increase in IC_50_ that would typify a resistant phenotype (17).

Subsequently, a panel of six lead antimalarial candidate compounds (Fig. 2B) was evaluated to determine their inhibitory effects on ABS parasites from the clinical isolates, compared to their efficacy against the reference strain PfNF54 (Fig. 2C). As indicated, these compounds were selected on the basis of their efficacy against ABS parasites and gametocytes as well as diversity in their evidenced drug targets and mode of action (9). All compounds displayed efficacy against PfNF54 within expected ranges as previously reported [Table S2 (13, 28, 40–43)], with acceptable dose-response curves obtained (Fig. S3). The PI4K inhibitor, MMV390048, was equally as effective against most clinical isolates compared to PfNF54 refence strain, with low-level (~2-fold) but significant increases in IC_50_ observed only against two isolates, SB1 and KF. This compound also produced bi-phasic IC_50_ curves against isolates SB3 and SB5 (Fig. S3), which could indicate that the parasite clones present within these isolates have differential drug sensitivity, with one or more clones less responsive to MMV390048 than the others. Other target-specific compounds, including the ATP4 inhibitors SJ733 and KAE609, were also able to inhibit ABS parasite proliferation in most clinical isolates (IC_50_ shifts <2-fold). However, for both compounds, a significant (*P* < 0.01, *t*-test, *n* = 3) loss in efficacy was observed against SB1 and SB4, against SB3; SJ733 at 6.8-fold less effective (IC_50_ of 170 nM compared to 25 nM for PfNF54, Table S2). This loss was even more pronounced with the pleiotropic compound, OZ439, that was unable to kill ABS parasites from clinical isolates SB1 and SB4, with an 11-fold loss in efficacy observed (Fig. 2C; Table S1). The translation elongation factor 2 (EF2) inhibitor M5717 remained effective against all isolates, with a detectable loss only observed against TD1, although potency was still retained at 0.71 nM vs 0.25 nM in NF54 (Fig. 2C; Table S2). Interestingly, this compound showed improved activity against ABS parasites from isolate SB10. As expected, the gametocyte-selective compound ML324 was unable to effectively inhibit ABS parasites from any of the clinical isolates (IC_50_ values >2.5 µM, Table S1). Together, this points to sustained efficacy of the antimalarial candidates against most clinical isolates, irrespective of target-specific or pleiotropic mode of action, with failure in efficacy (IC_50_ changes >5-fold) limited to isolates SB1, SB3, and SB4. The refractory nature of these isolates raises concerns that they may harbor non-responsive clones.

Pairwise testing for correlation between the *ex vivo* efficacy of the compounds was performed as an indication of shared responses between drugs on specific isolates, which may point to shared mechanisms explaining drug sensitivity profiles (Fig. 2D). The strongest positive correlations (Spearman’s rank coefficients of >0.5) were seen for chloroquine and amodiaquine (*r*_s_ = 0.90), similar to susceptibility correlations recently reported for clinical isolates from eastern Uganda (17) but also for chloroquine and artemisinin and DHA (*r*_s_ = 0.71 and 0.52). However, there was very little correlation between drug responses of the lead antimalarial candidates on the clinical isolates, with positive correlations (*r*_s_ ~0.39) only observed between SJ733 and KAE609, supporting the notion that they still have a shared target (PfATP4) in the clinical isolates. Interestingly, the pleiotropic compound OZ439 shared similar responses with the most potent compound M5717, as a protein synthesis inhibitor, and therefore the activity of both compounds in essential biological processes associated with protein metabolism is important to target clinical isolates. This was also extended to the lead antimalarial candidate MMV390048, with some positive correlation (>0.4) also observed between MQ and KAE609 (*r*_s_ 0.74) and OZ439 (*r*_s_ 0.68) (Fig. S4). Most of the lead antimalarial candidates showed anticorrelated activities on the clinical isolates in contrast to the known antimalarials, supporting novel mechanisms of actions and drug targets for these compounds.

### Lead antimalarial candidates show efficacy against *P. falciparum* gametocytes from clinical isolates

**Fig 3:**
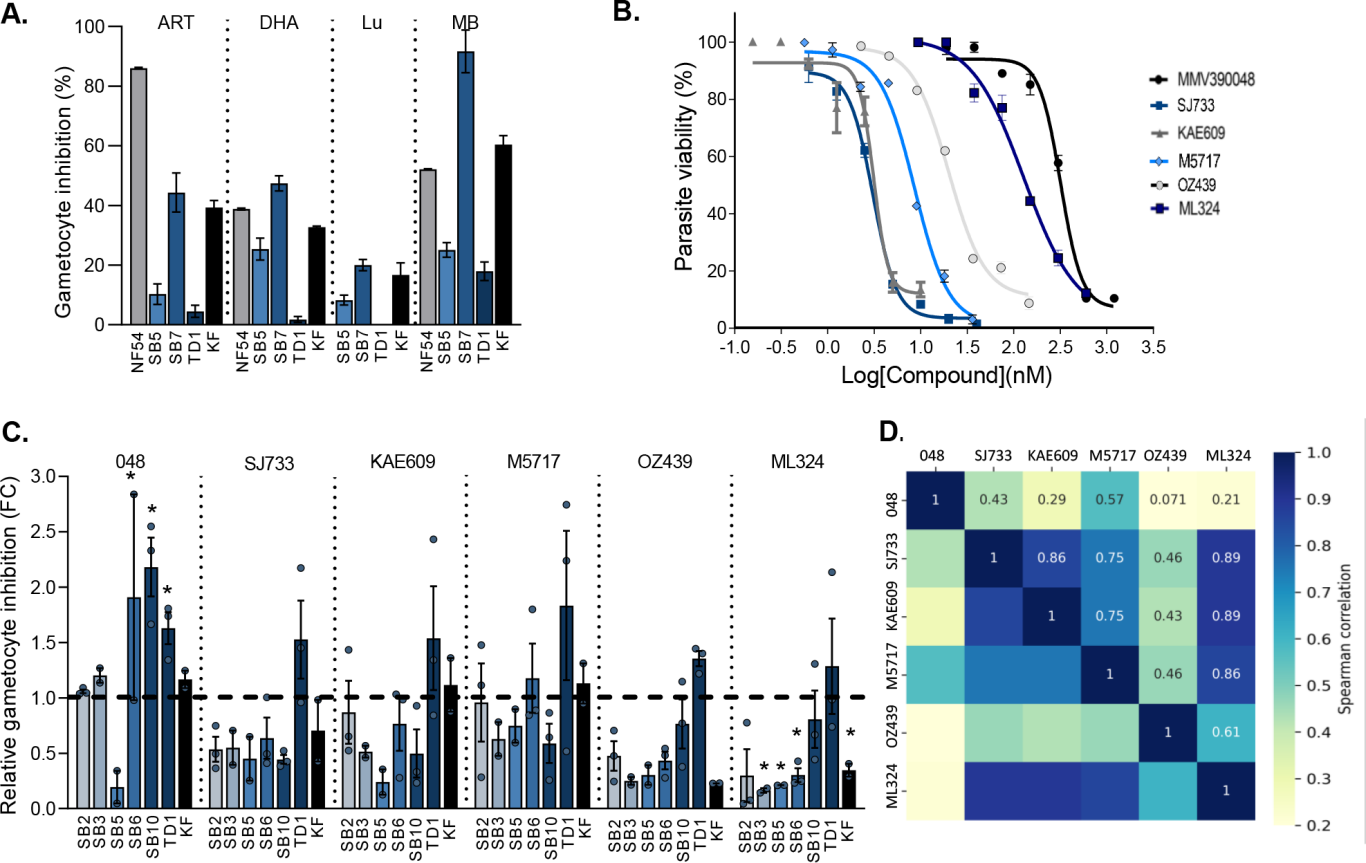
Gametocytocidal activity of antimalarial candidates against late-stage gametocytes from different isolates. (A). Gametocytocidal activity of standard antimalarials compounds against clinical isolates of *P. falciparum*. The pLDH assay was performed for each compound (1 µM) for a 48 h incubation against late-stage (IV and V) gametocytes. Data are from a single biological experiment, in technical triplicates ±SD. ART, DHA, lumefantrine (Lum), MB. (**B)** Dose-response curves for the lead antimalarial candidates against PfNF54 reference strain on the pLDH assay platform. Data are from three independent biological repeats, each in technical triplicates, mean ± standard error (S.E.), where not indicated; error bars fall within the symbol size. (**C)** Fold change in inhibition of gametocytocidal viability of lead antiplasmodial compounds against clinical isolates of *P. falciparum* compared to PfNF54. The pLDH assay was performed for each compound at 2× IC_50_ of NF54 reference strain (MMV390048: 230 nM, SJ733: 2 nM, KAE 609: 2 nM, M5717: 6 nM, OZ439: 7 nM, and ML324: 140 nM). Inhibition for each compound is indicated as relative to PfNF54 normalized at 1 as fold change (FC). Data are from three independent biological repeats, each performed in technical triplicates, mean ± S.E. Student’s *t*-test significance indicated as *P ≤ 0.05. (**D)** Pearson’s correlations between the activities (percent gametocyte inhibition) among the lead candidates. The magnitude and direction of the associations are indicated.

Based on the differential activity observed for some of the lead antimalarial compounds on ABS parasites of the clinical isolates, we subsequently sought to determine the ability of the compounds to target late-stage (stage IV or V) gametocytes produced from these isolates. First, we evaluated the performance of a few known antimalarials against late-stage gametocytes produced from a selected set of clinical isolates, SB5 and SB7 as moderate gametocyte producers, TD1 as a poor gametocyte producer, and KF as a high gametocyte producer (Fig. 3A). The activities obtained for artemisinin, DHA and MB [as antimalarials active against late-stage gametocytes, including stage IV gametocytes (10, 30)], and lumefantrine with poor gametocytocidal activity (44) were confirmed against PfNF54 (Fig. 3A) with reproducible assay performance indicators (*Z*-factor >0.8) as before (20, 22). ART, DHA, and MB had similar inhibition profiles, capable of targeting SB7 and KF gametocytes, but not as effective against gametocytes from TD1 and SB5. As expected, lumefantrine poorly targeted late-stage gametocytes from clinical isolates (Fig. 3A). The gametocytocidal activity of the six lead antimalarial candidates used in this study was confirmed against the PfNF54 reference strain, with the IC_50_ of the candidates corresponding in all cases with the reported values, ranging from potently active at ~1 nM to 230 nM (Fig. 3B; Table S3) (9, 10, 13, 24–27, 45).

The late-stage gametocytes from the clinical isolates were subsequently treated with the antimalarial candidates at 2× the IC_50_ determined against PfNF54 with the target-specific compounds MMV390048, M5717, KAE609, and SJ733 being the most potent gametocytocidal compounds. Most of the compounds were able to kill gametocytes from the clinical isolates to a similar extent as their ability to kill gametocytes from PfNF54 (<2-fold change in inhibition, Fig. 3C). Interestingly, MMV390048 showed a significantly improved ability to kill gametocytes from isolates SB6, TD1, and SB10. All the compounds retained gametocytocidal activity against TD1 as a relatively poor gametocyte producer, while only MMV390048 showed pronounced activity against SB10, one of the main gametocyte-producing isolates. By contrast, the protein-targeted compounds MMV390048, SJ733, KAE609, and M5717, but not the pleiotropic compound OZ439, retained some activity against the other high gametocyte producer KF, as well as against gametocytes from SB2 and SB6, two isolates with a proliferation preference with significantly lower levels of gametocytes produced.

Gametocytes produced from the SB3 and SB5 isolates were generally the most refractory to any gametocytocidal action, although these isolates produced viable stage IV and V gametocytes. The gametocyte-selective compound ML324 lost significant gametocytocidal activity against most of the isolates (Fig. 3C). Overall, SJ733 and KAE609 (as PfATP4 inhibitors) correlated in their activity profiles against the clinical isolates (*r*_s_ = 0.86, Fig. 3D) akin to the ability of M5717 to kill late-stage gametocytes (*r*_s_ of 0.75 against SJ733 and KAE609). Interestingly, this profile was also observed for ML324 (*r*_s_ of >0.85), suggesting that this compound could also have a specific target in gametocytes, whereas OZ439 as known pleiotropic compound had a profile different from all other compounds on the clinical isolates (Fig. 3D). The strong correlation between OZ439 and M5717 observed in ABS (*r*_s_ of 0.41, Fig. 2E) is mirrored in gametocytes (*r*_s_ of 0.46, Fig. 3D), suggesting that protein synthesis in gametocytes could be completely perturbed by specific inhibitors of protein synthesis (M5717) and protein alkylating agents (OZ439). These data are informative to translate target-specific actions of compounds, which are indicated in ABS stages, into their activity in gametocytes and confirms Achilles heels in gametocyte biology (9).

### Genotyping indicates parasite complexity and diversity

To understand the observed variation in susceptibility of different stages of *P. falciparum* clinical isolates to antimalarial compounds, we sought to determine the influence of genetic diversity within each isolate. First, we evaluated the presence of mutations that would confer drug resistance to known antimalarials in these isolates. Genes involved in drug resistance were sequenced to confirm the presence of specific mutations known to cause resistance to various antimalarial compounds (Table 1). This includes detection of mutations/increased transcript levels for the *P. falciparum* chloroquine resistance transporter (*pfcrt*), multidrug resistance transporter 1 (*pfmdr1*), dihydrofolate reductase (*pfdhfr*), and dihydropteroate synthase (*pfdhps*). The clinical isolates had mutations associated with *pfdhfr* and *pfdhps* (except for TD1) that mediate pyrimethamine, cycloguanil (46), and sulfadoxine resistance (47). Only five isolates had mutations associated with *pfcrt*, with a similar loss of such resistance markers seen in other countries in Africa (17). This is likely related to the discontinuation of chloroquine in southern Africa (48–50) and correlates to previous *in vitro* phenotypic data on the sensitivity of clinical isolates to chloroquine (18). The loss in efficacy of MQ and MB against the ABS parasites (Fig. 2A) could be due to the presence of mutations in *pfmdr1* in the SB4 isolate (Table 1). However, no other clear association could be identified between the drug resistance marker profiles of the clinical isolates and their susceptibility to known antimalarials shown here; clinical isolates SB1, SB3, and SB4 only show shared antifolate resistance, with only SB4 harboring changes in PfMDR1 expression, although a pronounced loss of drug efficacy was observed against these isolates.

**TABLE 1:** Origin and general drug resistance marker evaluation of the clinical isolates^*b*^

Clinical ID	Origin	Drug resistance markers
SB1	Unknown	pfdhfr, pfdhps (aF^R^)
SB2	Mozambique	pfdhfr, pfdhps (aF^R^)
SB3	Mozambique	pfdhfr, pfdhps (aF^R^)
SB4^*a*^	Malawi	pfdhfr, pfdhps, pfmdr1 (mixed, aF^R^)
SB5^*a*^	Mozambique	pfdhfr, pfdhps, pfcrt (aF^R^, CQ^R^)
SB6	Mozambique	pfdhfr, pfdhps, pfcrt (aF^R^, CQ^R^)
SB7^*a*^	Malawi	pfdhfr, pfdhps, pfcrt (aF^R^, CQ^R^)
SB10	Unknown	pfdhfr, pfdhps (aF^R^)
SB11	Unknown	pfdhfr, pfdhps, pfmdr1 (mixed, aF^R^)
TD1^*a*^	Mozambique	pfdhfr, pfcrt (aF^R^, CQ^R^)
TD2	Mozambique	pfdhfr, pfdhps, pfcrt (aF^R^, CQ^R^)
KF^*a*^	Mozambique	pfdhfr, pfdhps, pfcrt (aF^R^, CQ^R^)

^*a*^ Data from previous work (18) included here for comparison.

^*b*^ aF^R^, antifolate resistance; CQ^R^, chloroquine resistance; MDR, multidrug resistant mutations.

Since this loss in efficacy against these strains could therefore not be clearly correlated with known drug resistance markers, evaluating known drug resistance markers was therefore insufficient to explain the differential action of new antimalarial compounds as these markers are limiting in their focused nature on specific mutations. We subsequently employed genotyping tools to obtain an indication of genetic complexity in clinical isolates as an indicator of multiple strains, some of which may provide fitness benefits and cause differences in drug susceptibility (39, 51). MS markers (31, 32, 34, 52) have been successfully used to determine genetic relatedness between parasites, population structure, and dynamics of clinical isolates from different transmission settings (35, 53, 54). The panel of 26 MS markers (31, 32) includes *pfg377*, a polymorphic gametocyte-specific antigen. This allows the study of the parasite clones that are actually transmitted (55), as diversity at this site in the genome is associated with adaptive survival mechanisms due to reduced transmission (56, 57).

**Fig 4:**
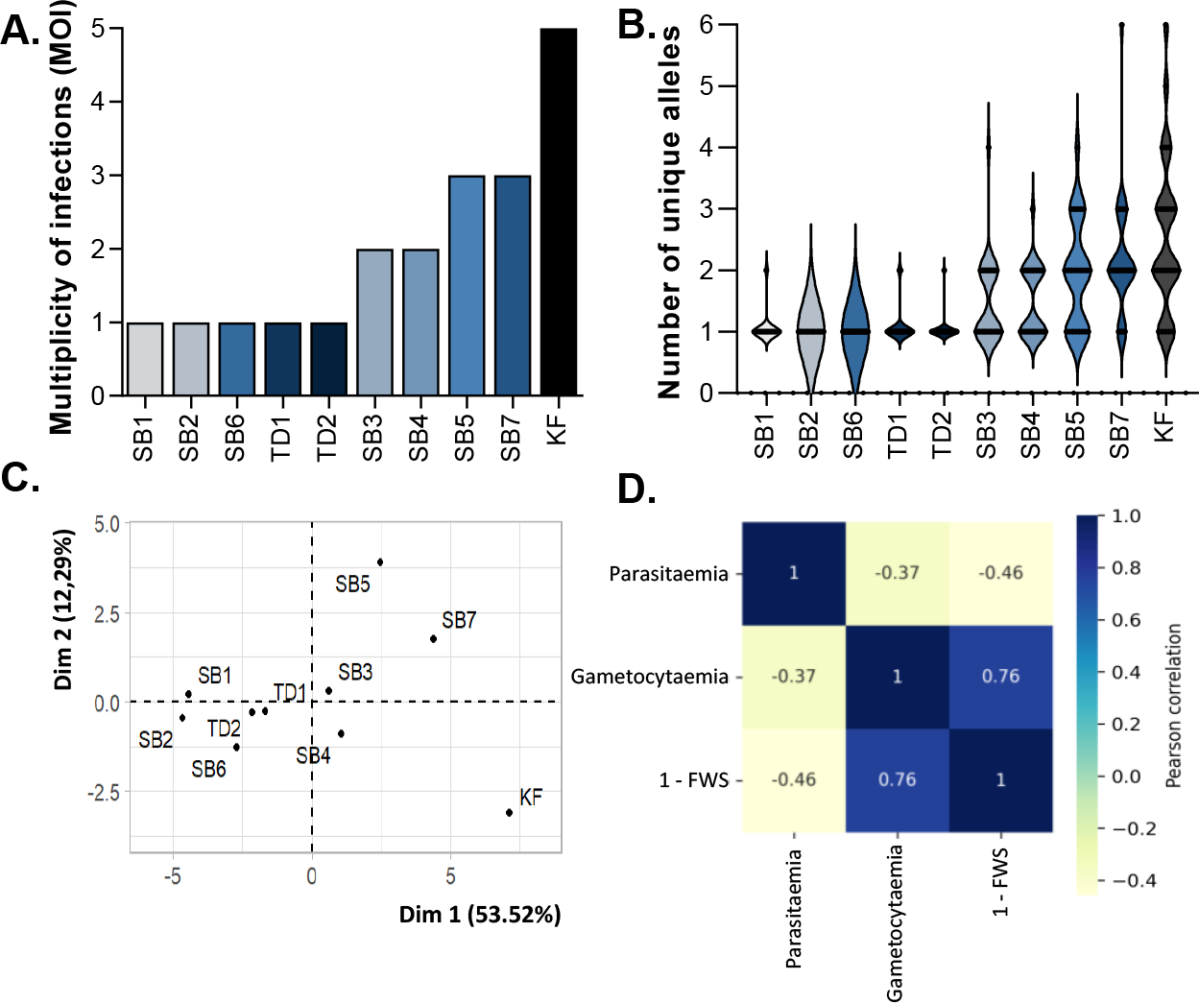
MS-based genotyping of the *P. falciparum* field isolates. MOI as contributed by alleles detected on various loci. (**A)** MOI was measured as the second-highest number of alleles detected at any of the 26 loci. (**B)** Violin plots showing full distribution of the data of the number of unique alleles for each clinical isolate. (**C)** Multiple component analyses of the contribution of alleles in each field isolate to variability in the population. (**D)** Pearson’s correlation coefficients as a correlation plot for the association between % parasitemia and % gametocytemia of the *ex vivo* cultures at the point of evaluation of drug efficacy and the genetic complexity of the field isolates as indicated by 1-Fws indices.

Of the clinical isolates, 10 samples had sufficient coverage at a minimum of 15 of the 26 MS loci evaluated. Half of the isolates were characterized as polyclonal infections (MOI ≥2), with an overall mean MOI = 2 for all the isolates (Fig. 4A), similar to multiple-clone *P. falciparum* infections previously reported for sub-Saharan Africa (58) and an indication of ongoing transmission in the sampling area in southern Africa. The isolates SB1, SB2, SB6, TD1, and TD2 each had an MOI = 1, indicative of the least amount of genetic complexity within each of these isolates. KF was the most genetically complex isolate with a MOI = 5 (Fig. 4A; Table S4). Isolates SB5 and SB7 each had an MOI = 3, and the clones within these two isolates were unique as they did not share any alleles (Fig. 4B). MCA (59) confirmed the observed distinction between the clinical isolates (Fig. 4C), with isolates SB1, SB2, SB6, TD1, and TD2 genetically more related to each other, clustering together, and contributing between 5% and 15% of the overall genetic variance in all the isolates. SB5, SB7, and KF are not only the most complex isolates but also distinct, with polymorphisms mostly present in unique MS loci compared to the other isolates. The multiclonal isolates only share polymorphisms at three MS loci, AS12, AS15, and AS31, with only A12 present in the coding region of a nucleoporin protein [PF3D7_0609000 (60, 61)], with A15 and AS31 in the untranslated region of proteins with unknown function (PF3D7_1364400 and PF3D7_0619100).

The variation in genetic complexity between isolates was supported by the observed level of within-host genetic diversity of each isolate as described by the Fws index (the within-host infection fixation index), which revealed that the probability of recombination of different clones was lowest in SB2 and SB6 (Fws at 1 indicative of a clonal infection) due to the low complexity of infection. Isolates SB3, SB4, SB5, SB7, and KF were all classified as highly diverse based on Fws of ≤0.70 with Fws values of 0.69, 0.67, 0.53, 0.41, and 0.30 from least to most diverse, respectively (Table S5). This heterogeneity could also be specifically associated with the polymorphic *pfg377* gametocyte marker (55), where both KF and SB7 were the only isolates for which low Fws indices of 0.013 and 0.26, respectively, were indicated for this allele. Isolates TD1 and TD2, SB1, SB2, and SB6 were all clonal with Fws of ≥0.95.

Genetic complexity showed an overall anticorrelated relationship with the ability of the parasites from clinical isolates to replicate, with ABS parasite replication increasing in genetically more clonal parasites (Pearson’s *r* = −0.46, Fig. 4D). By contrast, genetically complex clinical isolates produce more gametocytes with a significant correlation between genetic complexity and gametocytemia (Pearson’s *r* = 0.76, *P* = 0.004; Fig. 4D), and therefore, parasites from these more genetically diverse infections appear to commit a higher proportion of parasites to reproduction above replication.

### Genetic complexity associates with antimalarial compound efficacy

**Fig 5:**
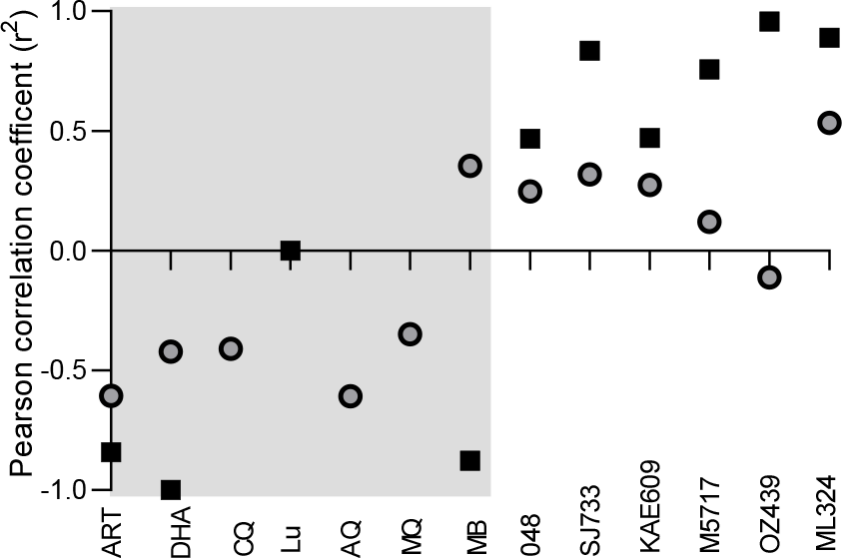
Genetic complexity associates with antimalarial compound efficacy. Pearson’s correlation between the genetic complexity (1-Fws indices) compared to compound activity both for ABS parasites (gray circles) and gametocytes (black squares). In each instance, the lines fitted are linear regressions as a visual indicator of correlation only. Gray and white blocks indicate known and lead antimalarials, respectively.

We next sought to determine if there was any relationship between the genetic complexity, as indicated by MS analyses of the *P. falciparum* clinical isolates and their susceptibility to chemical interference, both for ABS parasite forms and gametocytes produced from the clinical isolates. Pearson’s correlation analyses between the ABS parasites’ susceptibility (IC_50_ values) and that of the within-host genetic diversity (1-Fws index, with 1 = genetically diverse and 0 = clonal), indicated a slightly positive association between the loss of efficacy (increased IC_50_ values) of the lead antimalarial candidates and the genetic complexity of the clinical isolates (increased 1-Fws indices, Fig. 5). This may suggest that these compounds preferentially kill ABS parasites from more clonal isolates, although they can also target ABS parasites from multiclonal isolates, given the rather low association (Pearson’s *r* between 0.1 and 0.3). However, the known antimalarials all show a strong negative correlation (*r* <−0.5), indicating that these compounds can effectively target ABS parasites from multiclonal isolates.

The association between drug efficacy and genetic diversity is particularly pronounced for the gametocytocidal activity of the compounds. Strong positive correlations (*r* >0.5) were observed for most of the lead antimalarial candidates (Fig. 5), with increased gametocyte viability (indication of compound inactivity) correlated with genetic complexity (high 1-Fws values). In essence, these compounds were only able to kill gametocytes originating from more clonal isolates such as those from SB2, SB6, and TD1. MB activity is anticorrelated with genetic complexity, and this compound fares much better against genetically diverse isolates compared to clonal isolates. Interestingly, the activity of known antimalarials did not show the same pronounced relationship, with particularly the artemisinins capable of targeting both ABS parasites and gametocytes from clonal as well as genetically complex clinical isolates (*r* <−0.5, Fig. 5). This points to the fact that compounds (e.g., MB, ART, and DHA) with more pleiotropic action against multiple essential biological processes in the parasite can more easily target refractory gametocytes from multiple-clone infections.

## DISCUSSION

Determining the efficacy of lead antimalarial candidates against relevant clinical isolates remains key to progress these during (pre)-clinical investigations. Although this is routinely performed for compounds with activity against ABS parasites, the gametocytocidal activity (as a proxy for transmission-blocking ability of a compound) of highly sought after compounds with activity against multiple stages is not determined. Additionally, no systematic investigations have been conducted that link parasite genetic complexity with differential efficacy of antimalarial agents against *P. falciparum* clinical isolates, which limits interpretations of the consequence of loss of activity. Here, we present an analysis of the activities of front-runner antimalarial candidates on both ABS parasites and gametocytes from clinical isolates of *P. falciparum* and connect these activities with genetic diversity within these isolates to provide explanations for differential antimalarial capacity.

The *P. falciparum* clinical isolates used here from southern Africa are attuned to either replication or reproduction, with an anticorrelated relationship observed in the sample set. Such preferences for either replication or reproduction have been seen also on a population level associated with changes in environmental conditions (62, 63) and within-host factors including declining immunity (64) and nutritional depletion (65–67). As a result, reproduction (as increased gametocyte prevalence) is prioritized to ensure transmission efficiency (68). This is pronounced in low-transmission settings (62), when parasite prevalence decreases to switch parasites to a reproductive profile, potentially as a result of within-host competition as has been reported for West African parasite populations (69), exacerbated by intrahost dynamics (53). The correlation of these observations with *in vivo* reports provides confidence that the observed phenotypes are a direct reflection of the complexity of gametocyte production and genotypes in the sample set.

Our data indicate a clear correlation between the ability of clinical isolates to produce gametocytes (as measured by *ex vivo* gametocytemias) and the genetic complexity within the samples (as evidenced from MS analyses), with multiple clonality and genetic diversity associated with increased gametocytogenesis. Increased diversity has been associated with higher transmission dynamics in West Africa (70) with higher recombination between multiple clones present, leading to increased population and within-host genetic diversity of the ABS parasites (35) and gametocytes (71). This has also been reported in low-transmission areas in southern Africa, where high genetic diversity is present due to imported cases from neighboring high-transmission countries (32, 72). However, the transmission dynamics in these reports were associated with total parasite prevalence and did not measure gametocyte prevalence exclusively due to the concomitant presence of asexual parasites. It would therefore be important to extend our findings to a population level to associate *in vivo* gametocytemia [e.g., by molecular marker detection of gametocytes (73)] with genetic complexity. Such analyses would require additional polymorphic markers to understand the differential contribution of clones to gametocyte production. Our data suggest that this is possible since the gametocyte-specific polymorphic marker *pfg377* (55) was clearly associated with high genetic complexity and gametocyte densities in the clinical isolates. It would also be of interest to understand the mechanisms for increased gametocytogenesis observed in some of the clinical isolates, including testing for levels of *Pfap2g* [as molecular transcriptional switch (74)], *gdv1*, and HP1 (75). Recently, within-host competition has been put forward to explain that ABS parasites’ growth phenotypes are essentially dictated by the behavior of the most prominent and successful clone (39). This information is not known for gametocytogenesis and, therefore, subcloning of the clinical isolates used here would be required to observe if this logic can be extended to associate increased gametocyte production with specific dominant clonal lineages within a multiple-clone infection.

Our data importantly indicate a relationship between increased propensity toward gametocytogenesis, parasite genetic diversity, and the efficacy of antimalarial compounds. Most of the antimalarial compounds tested here were active against ABS parasites from both clonal and multiclonal parasites at concentrations comparable to reference strains and appropriate for clinical application. However, our data suggest a specific relationship between genetic diversity in clinical isolates and their sensitivity to antimalarial compounds, where some antimalarial compounds show loss of efficacy against genetically more complex isolates, a phenotype that is particularly pronounced in gametocytes. Therefore the compounds tested here were not as effective against gametocytes produced from multiclonal parasites compared to clonal ones, except for compounds with more pleiotropic actions, supporting the notion that compounds acting supposedly on multiple targets may be effective gametocytocidal tools (8). This suggests that increasing genetic complexity provides a fitness benefit to more genetically diverse gametocytes that result in one (or more) superior clone fitter for transmission. However, this also translates to these clones being more refractory to gametocytocidal action.

The differential activity associated with genetic complexity is independent of the genetic diversity described through analysis of known drug resistance markers. There are limited rigorous analyses that associate genetic complexity with drug susceptibility beyond several reports that investigate drug resistance markers as a proxy of genetic diversity in clinical isolates [e.g., references (16, 17)]. As far as we know, this is the first report to associate genetic diversity (beyond known drug resistance markers) with drug susceptibility in clinical isolates in Africa, for both ABS parasites and gametocytes. It remains to be seen whether this is the case *in vivo* with a larger longitudinal study and what molecular factors contribute to this potential survival advantage in gametocytes from multiclonal isolates. Our data are also not able to inform whether the loss in efficacy is indicative of a functionally resistant phenotype to certain antimalarial classes or if polymorphisms exist in the specific drug targets associated with the candidate drugs (16). Since dominant clones in a multiclonal infection can dominate an ABS parasite growth phenotype (39), it would be important to identify such clones through subcloning, followed by in-depth genomic evaluations to allow for the association of causality with loss of gametocytocidal efficacy, as has been done for ABS parasites.

Our findings therefore suggest that multistage active antimalarials could lose gametocytocidal efficacy in multiple-clone infections, which is typically associated with high-transmission settings. This would imply that transmission-blocking strategies would be more useful in low-transmission settings where more clonal parasite populations are present. This would result in effective targeting of gametocytes if the reported increased gametocyte densities in these settings can be targeted within the exposure concentration window of drug administered to patients. In high-transmission settings, genetic diversity is high, but lower gametocytemias have been reported, and it remains to be seen if drugs are active against these lower gametocyte densities even in the presence of genetic diversity. Additionally, it is not clear if the decrease in drug activity will be able to overcome potential outperforming clones in a multiclone infection. Monitoring genetic diversity in gametocytes is key, since within-host competition between clones (resulting in higher gametocyte carriages in populations) could result in highly dominant gametocytes that are not only present at high levels but also more refractory to antimalarial compounds. This would ensure sustained transmission of the parasites and challenge the efficacy of transmission-blocking interventions.

## Data Availability

The data sets supporting the conclusions of this article are available from the corresponding authors on reasonable request

## SUPPLEMENTAL MATERIAL

Supplementary Fig S1-S4, Tables S1-S5 aac.01291-23-s0001.pdf Supplementary figures and tables.

ASM does not own the copyrights to Supplemental Material that may be linked to, or accessed through, an article. The authors have granted ASM a non-exclusive, world-wide license to publish the Supplemental Material files. Please contact the corresponding author directly for reuse.
