# Supplementary material for: Genetic complexity alters drug susceptibility of asexual and gametocyte stages of Plasmodium falciparum to antimalarial candidates
Source: Antimicrob Agents Chemother. 2024 Jan 23;68(3):e01291-23. doi: 10.1128/aac.01291-23 (PMC10916389; doi:10.1128/aac.01291-23)
Supplement: Supplementary Fig S1-S4, Tables S1-S5 — Supplementary figures and tables. [file aac.01291-23-s0001.pdf]

Supplementary Material

1 Supplementary figures

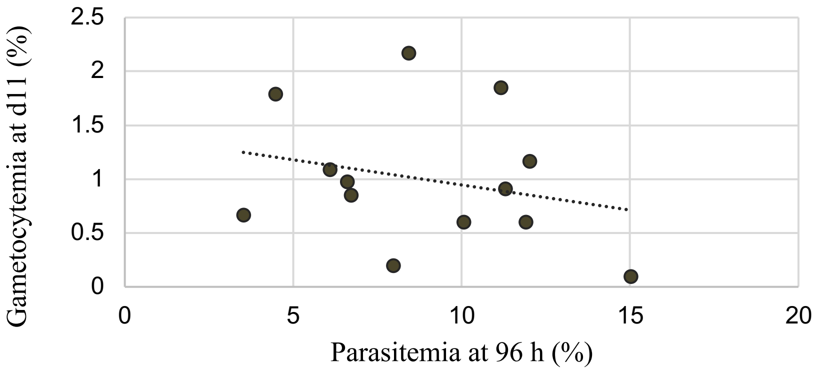

Figure S1: Anticorrelated relationship between parasitaemia and gametocytaemia

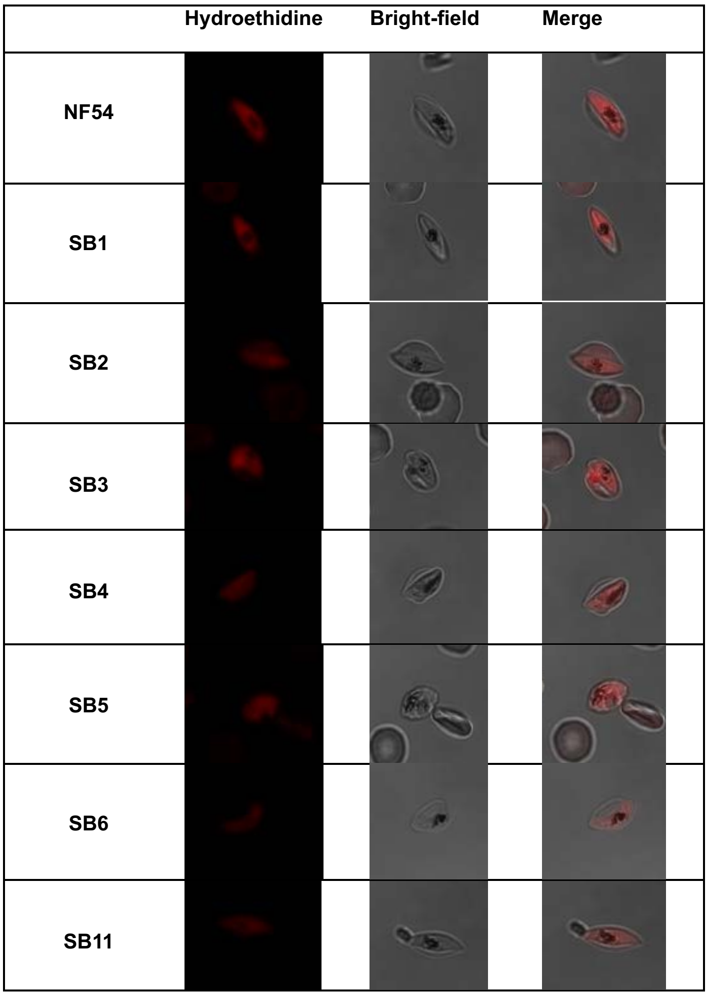

Figure S2: Confocal images of late-stage gametocytes from the isolates, stained with hydroethidine as a proxy of viability 100x magnification used.

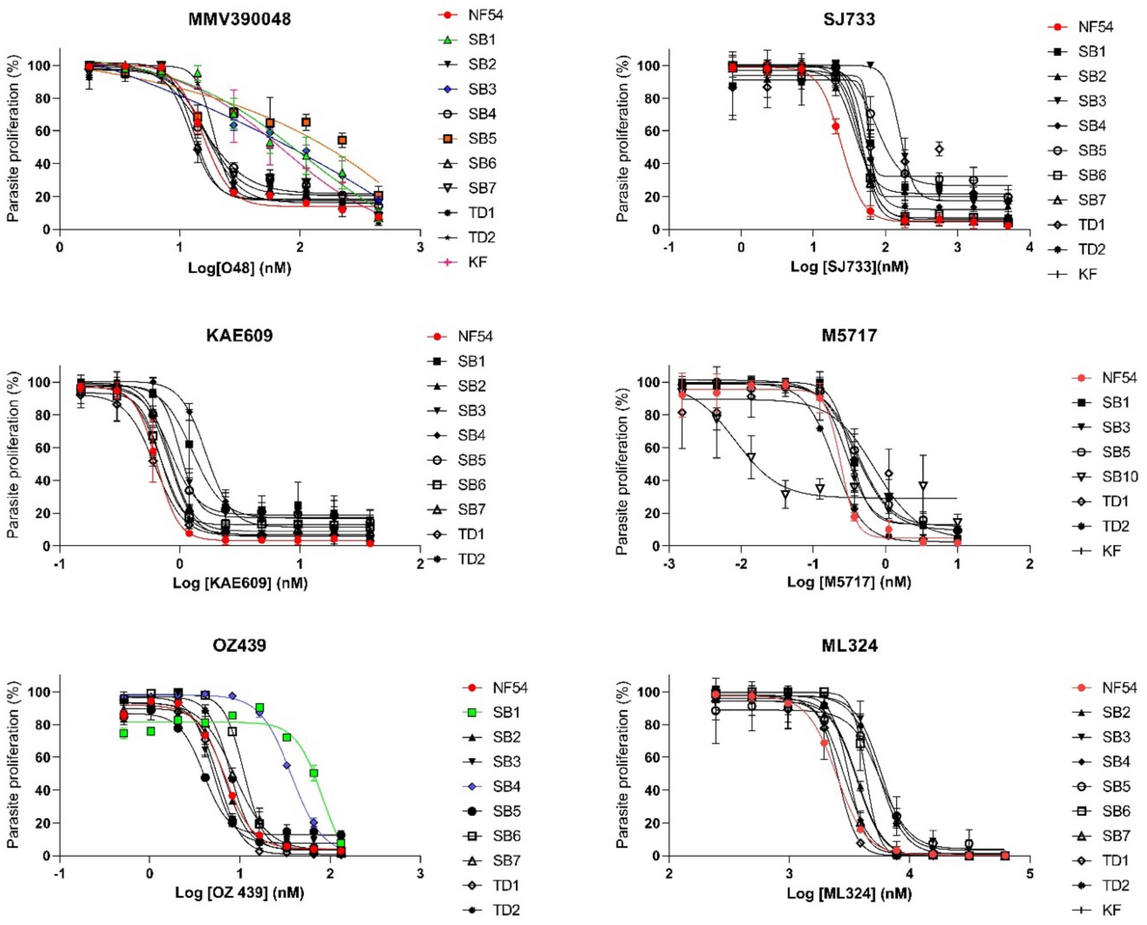

Figure S3: IC_{50} curves of ABS parasites from the isolates compared to NF54 (in red) treated with the different compounds.

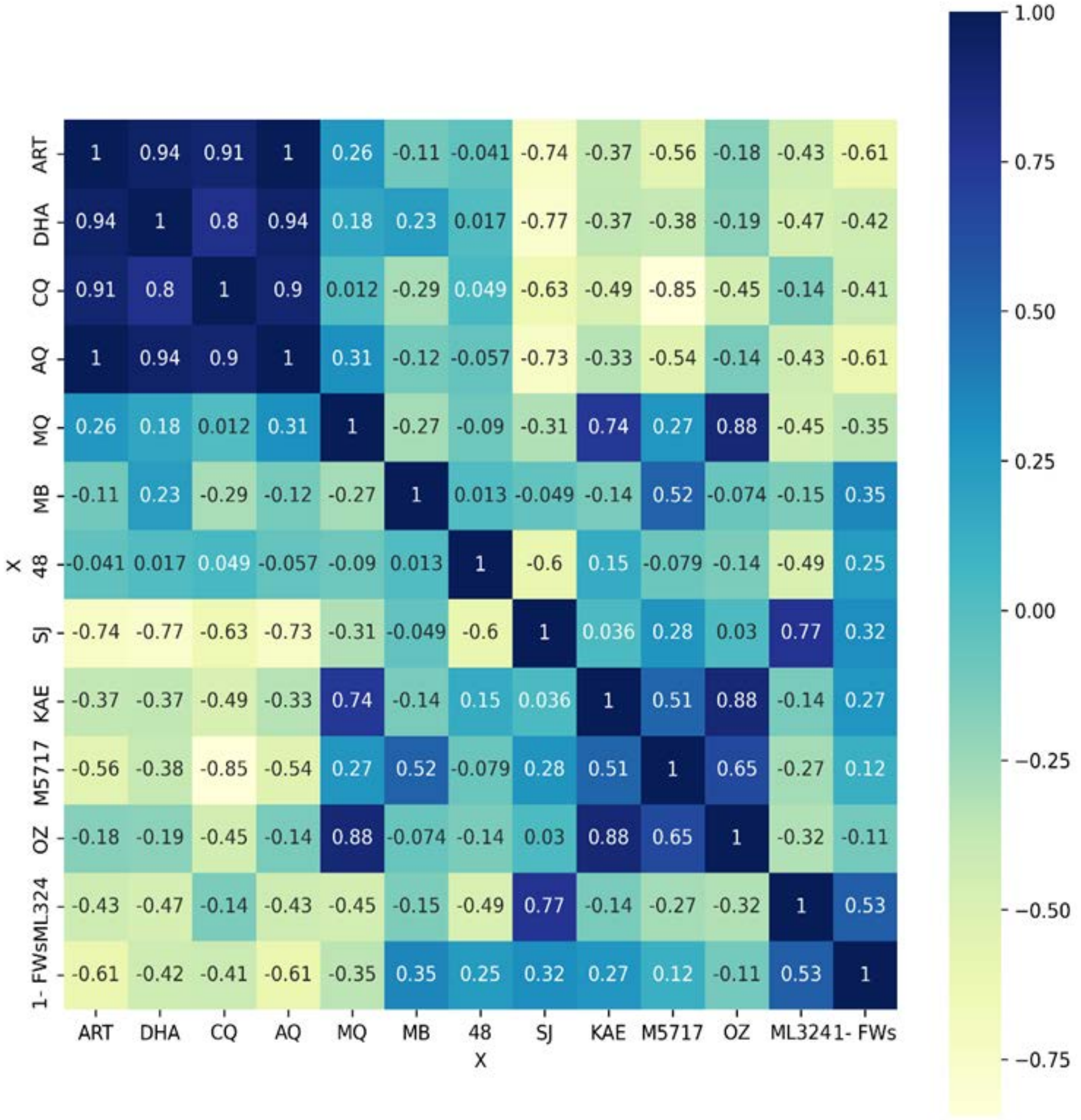

Figure S4: Correlation of drug efficacy between isolates across all isolates, for known antimalarial candidates as well as lead antimalarials.

2 Supplementary table

Table S2: IC_{50} values of known antimalarial compounds against ABS parasites from PfNF54 and selected clinical isolates.

IC₅₀ values (nM; avg + S.E.)						
	**ART**	**DHA**	**CQ**	**AQ**	**MQ**	**MB**
**NF54**	6.5	2.1	6.8	3.0	20.0	21.9
**SB4**	0.38 + 0.28	1	2.32	0.79 + 0.12	85.78 + 5.90	1.33 + 0.33
**SB5**	3.14 + 0.21	1	3.93 + 0.07	2.34 + 0.17	3.69 + 0.32	1.33 + 0.3
**SB7**	3.16 + 0.40	1.25 + 0.47	3.48 + 0.16	2.09 + 0.03	7.37 + 0.55	63.31 + 11.37
**TD1**	6.23 + 0.25	2.38 + 0.18	5.46 + 0.31	4.49 + 0.04	10.04 + 1.21	3.67 + 0.13
**KF**	3.75 + 0.05	2.16 + 0.01	ND	4.83 + 0.44	4.6 + 2.36	1

ND = not determined

IC_{50} values are from three independent biological repeats, each performed in technical triplicates, average

Artemisinin (ART), dihydroartemisinin (DHA), chloroquine (CHQ), amodiaquine (ADQ), mefloquine (MQ) and methylene Blue (MB)

Table S2: IC_{50} values of lead antimalarial candidate compounds against ABS parasites from PfNF54 and clinical isolates.

IC₅₀ values (nM; avg + S.E.)						
	**MMV390048**	**SJ733**	**KAE609**	**M5717**	**OZ439**	**ML324[a]**
**NF54**	15.57 + 1.10	25.08 + 0.60	0.65 + 0.05	0.25 + 0.02	6.84 + 0.15	2.47 + 0.03
**SB1**	36.35 + 1.55	61.28 + 0.47	1.26 + 0.19	0.43 + 0.12	75.4 + 7.34	2.61 + 0.01
**SB2**	12.47 + 0.53	37.51 + 3.60	0.70 + 0.05	ND	6.84+ 0.20	3.65 + 0.02
**SB3**	ND	170.33 + 6.84	1.00 + 0.04	0.28 + 0.05	4.94 + 0.09	5.81 + 0.08
**SB4**	15.32 + 1.01	52.82 + 7.45	1.66	ND	37.51 + 2.94	2.92 + 0.16
**SB5**	ND	77.16 + 12.33	0.72 + 0.19	0.42 + 0.05	3.96 + 0.29	5.66 + 0.38
**SB6**	13.07 + 0.18	47.9 + 10.38	0.68 + 0.04	ND	10.90 + 0.91	4.16 + 0.14
**SB7**	17.17 + 1.56	50.99 + 4.98	0.82 + 0.04	ND	8.83+ 0.54	3.22 + 0.24
**SB10**	ND	ND	0.45 + 0.09	0.02 + 0.01	ND	ND
**TD1**	19.02 + 5.06	55.12 + 1.86	0.61 + 0.02	0.71 + 0.21	5.01 + 0.22	2.81 + 0.43
**TD2**	18.96 + 0.80	43.45 + 2.99	0.82 + 0.02	0.19 + 0.01	7.83 + 0.07	5.51 + 0.09
**KF**	32.75 + 0.82	42.08 + 3.67	ND	0.32 + 0.03	ND	3.81 + 0.66

[a] Data for ML324 is reported in μM

ND = not determined

IC_{50} values are from three independent biological repeats, each performed in technical triplicates, average with S.E.

Table S3: Activity of frontrunner antimalarials against late-stage PfNF54 gametocytes determined using pLDH assay, compared to published values.

**Compound**	**IC₅₀ + S.E. (nM)**	**Published IC₅₀**	**Reference**
MMV390048	230.70 + 37.37	214 nM (stage II/III)	(Paquet et al., 2017)
SJ733	1.11 + 0.49	5 mg/kg (ED₅₀ in *P. berghei*) [a]	(Dechering et al., 2017)
KAE609	1.08 + 0.38	100 (reducing transmission at 5 μM)	(van Pelt-Koops et al., 2012)
M5717	3.10 + 0.34	2 nM (stage IV)	(Baragaña et al., 2015, Plouffe et al., 2016)
OZ439	3.62 + 0.41	2 nM (stage II/III)	(Giannangelo et al., 2020)
ML324	70.37 + 3.80	77 nM (stage V)	(Reader et al., 2021)

[a] No *P. falciparum* data available

Table S4: Microsatellite markers used in study with the unique number of alleles identified for each isolate's MOI.

Isolate	Ara2	AS1	AS11	AS12	AS14	AS15	AS19	AS2	AS21	AS25	AS3	AS31	AS32
SB1	0	0	1	0	1	1	0	1	1	0	0	1	1
SB2	0	0	1	0	1	1	0	1	0	0	1	0	1
SB3	2	1	2	2	1	2	2	1	0	4	1	2	1
SB4	2	1	1	2	2	2	2	1	0	1	2	2	1
SB5	3	1	1	2	4	2	1	2	0	3	1	2	3
SB6	1	1	1	1	1	1	1	1	0	1	1	1	1
SB7	2	1	2	2	2	2	2	3	2	6	3	3	1
TD1	1	1	1	1	1	1	1	1	0	1	1	1	1
TD2	1	1	1	1	1	1	1	1	0	1	1	1	1
KF	3	2	4	3	6	2	3	2	2	2	3	4	2

Isolate	AS34	AS8	B7M19	PFG377	TA109	TA40	TA60	TA81	TA87	AS7	PolyA	PfPK2
SB1	1	1	0	1	0	0	1	0	0	1	2	0
SB2	1	1	0	1	0	0	1	0	0	1	1	0
SB3	2	2	1	1	2	1	1	1	1	1	1	0
SB4	1	1	1	1	1	1	3	2	2	2	1	0
SB5	1	2	2	1	2	2	1	3	1	2	3	0
SB6	1	1	0	0	1	1	1	1	1	1	1	1
SB7	1	3	2	2	3	2	2	2	1	1	2	0
TD1	1	1	1	1	1	1	1	1	2	1	2	0
TD2	1	1	1	1	1	1	1	1	1	1	2	1
KF	1	1	1	3	3	5	2	4	2	1	1	0

Table S5: F_{WS} indices for the clinical isolates.

**Isolate**	SB1	SB2	SB3	SB4	SB5	SB6	SB7	TD1	TD2	KF
**F_{WS}**	0.959	1	0.690	0.672	0.533	1	0.413	0.953	0.977	0.302

*F_{WS} was calculated for each sample using equation $F_{WS} = 1 - Hw/Hs$, where *Hw* is the allele frequency of each unique allele found at a particular locus for each individual and *Hs* is the heterozygosity of the local parasite population.
